# Machine Learning-Based Classification of BI-RADS 4 and BI-RADS 5 Microcalcifications in Mammography Combined with DCE-MRI for Malignant–Benign Discrimination

**DOI:** 10.3390/tomography12060088

**Published:** 2026-06-17

**Authors:** Sevgi Ünal, Enes Açıkgözoğlu

**Affiliations:** 1 Department of Radiology, Izmir Katip Celebi University Ataturk Training and Research Hospital, Izmir 35360, Turkey; 2 Cloud Computing Operators, Isparta School of Information Technologies, Isparta University of Applied Sciences, Isparta 32200, Turkey; enesacikgozoglu@isparta.edu.tr

**Keywords:** breast cancer, mammography, microcalcifications, BI-RADS classification, machine learning, medical image analysis, computer-aided diagnosis

## Abstract

Background/Objectives: Breast cancer remains one of the leading causes of cancer-related mortality among women worldwide. Early and accurate characterization of suspicious mammographic microcalcifications is essential for improving diagnostic decision-making and reducing unnecessary invasive procedures. Microcalcifications classified as BI-RADS 4 and BI-RADS 5 are clinically important radiological findings; however, differentiating benign from malignant lesions remains challenging because of overlapping morphological and distribution patterns. This study aimed to develop a structured feature-based machine learning model for predicting the pathological diagnosis of breast microcalcifications by integrating mammographic descriptors, patient age, and dynamic contrast-enhanced magnetic resonance imaging (DCE-MRI) contrast enhancement findings. Methods: The dataset included 53 biopsy-confirmed cases and consisted of clinical and radiological variables, including patient age, calcification morphology, calcification size, distribution pattern, DCE-MRI contrast enhancement status, and histopathological outcome. Several conventional machine learning algorithms were evaluated, including Logistic Regression, Support Vector Machine with radial basis function kernel, K-Nearest Neighbors, Decision Tree, Random Forest, Extra Trees, Gradient Boosting, AdaBoost, and CatBoost. Hyperparameter optimization was performed using grid search with five-fold cross-validation. Model performance was assessed using accuracy, precision, recall, F1-score, ROC-AUC, and log loss. Results: Logistic Regression achieved the highest overall performance, with an accuracy of 0.909 and an F1-score of 0.889, while AdaBoost achieved a recall of 1.000 in the internal evaluation. However, given the limited sample size and lack of external validation, these findings should be interpreted as preliminary. Conclusions: The results suggest that structured radiological descriptors combined with DCE-MRI enhancement information may support malignancy risk stratification of BI-RADS 4–5 microcalcifications, although larger multicenter studies are required before clinical implementation.

## Simple Summary

Suspicious microcalcifications seen on mammography are common findings that often require biopsy because distinguishing benign from malignant cases can be difficult. This study evaluated whether routinely available mammographic descriptors, patient age, and DCE-MRI contrast enhancement findings could be combined in a machine learning model to support malignancy prediction for BI-RADS 4–5 microcalcifications. Among the tested models, Logistic Regression showed the highest internal performance. However, the dataset was small and from a single center, so the results should be considered preliminary and require validation in larger multicenter studies before clinical use.

## 1. Introduction

Breast cancer is the most commonly diagnosed cancer in women worldwide and the second leading cause of cancer-related deaths [1]. Mammography is an imaging method used for breast cancer screening and early diagnosis. It has been proven that mammography screening can reduce breast cancer mortality by up to 30% [2]. Breast microcalcifications are one of the early signs of breast cancer and can be detected as the sole finding in 30–50% of breast cancers during screenings [3].

According to the fifth edition of the Breast Imaging Reporting and Data System (BI-RADS), updated in 2013, biopsy is recommended for suspicious microcalcifications classified as BI-RADS categories 4 and 5. The evaluation of microcalcifications is heterogeneous; this is likely due to the wide range of patient subgroups (BI-RADS 4a, 4b, and 4c) [4]. However, the positive predictive value (PPV) of microcalcifications for malignancy ranges from 15.9% to 90.6%, covering a wide range consistent with BI-RADS [5]. Although microcalcifications detected on mammography are often the sole finding associated with ductal carcinoma in situ (DCIS), most are benign [6].

There is significant variability among radiologists, and the positive predictive value (PPV) for mammographic microcalcifications generally does not exceed 30%; sensitivity ranges from 66.7% to 98.6%, while specificity ranges from 71.2% to 96.9% [7]. Today, characterizing suspicious microcalcifications on mammograms based on clinical factors and mammographic features remains a challenging process for radiologists. This situation leads to unnecessary biopsies, increased costs, and heightened anxiety among patients [8]. There is a need for an alternative imaging method to more accurately distinguish between benign and malignant microcalcifications. According to a meta-regression analysis, the presence of contrast uptake on dynamic contrast-enhanced MRI (DCE-MRI) is the diagnostic criterion associated with the highest sensitivity in the diagnosis of malignancy (*p* ≤ 0.013) [9]. Recent meta-analyses suggest that the absence of suspicious contrast enhancement on DCE-MRI may be associated with a lower probability of malignancy in selected BI-RADS 4 microcalcifications and may help guide biopsy decision-making. However, absence of contrast enhancement should not be interpreted as independently excluding malignancy, particularly when DCE-MRI assessment is simplified into a binary enhancement variable [9]. In a study evaluating the sensitivity of DCE-MRI for amorphous calcifications, it was emphasized that DCE-MRI demonstrates high sensitivity and reduces unnecessary biopsies [10].

Wang et al. demonstrated that deep learning approaches can effectively differentiate benign and malignant breast lesions containing microcalcifications on mammograms, reporting promising classification performance and highlighting the feasibility of automated data-driven assessment in breast imaging. Their findings suggested that deep models may capture subtle discriminative patterns that are difficult to quantify through conventional visual interpretation alone, thereby supporting the use of artificial intelligence in the analysis of suspicious calcifications [11].

More recently, Kang et al. evaluated several transfer learning-based deep convolutional neural network models for the classification of suspicious microcalcifications detected on screening mammography. Their results showed that ensemble and high-capacity pretrained models achieved strong diagnostic performance, while visual explanation maps confirmed that the networks focused on clinically relevant calcification regions. These findings further support the potential of deep learning systems as decision-support tools for reducing unnecessary biopsies in suspicious mammographic findings [12].

In addition, Meng et al. investigated deep transfer learning in BI-RADS 4 mammographic lesions and showed that artificial intelligence-based models may contribute to biopsy decision support, particularly in challenging indeterminate cases. Although their work was not limited specifically to microcalcifications, it reinforced the broader clinical value of AI-assisted malignancy prediction in BI-RADS 4 lesions and emphasized the role of model-based assessment in improving diagnostic confidence and reducing unnecessary invasive procedures [13].

Recent studies have further emphasized the diagnostic relevance of mammographic microcalcification morphology and distribution patterns in relation to histopathological outcomes. Specific descriptors such as heterogeneous morphology, fine linear or branching pleomorphic microcalcifications, and linear or segmental distribution patterns have been associated with a higher positive predictive value for malignancy and an increased risk of ductal carcinoma in situ or invasive carcinoma [14]. These findings support the continued clinical importance of BI-RADS-based morphological descriptors in malignancy risk stratification, particularly for suspicious calcifications that require biopsy decision-making [14]. In parallel, recent artificial intelligence studies have attempted to improve the detection and classification of mammographic microcalcifications. AI-assisted digital mammography has been reported to enhance the sensitivity of suspicious grouped microcalcification detection, although expert radiologist interpretation remains necessary for appropriate clinical specification [15]. Similarly, large-scale deep learning pipelines have been developed for mammographic microcalcification detection and benign–malignant classification, demonstrating the potential of automated image-based systems to support radiological assessment [16]. EfficientNet-based convolutional neural networks have also shown strong performance in classifying benign and malignant mammographic calcifications, highlighting the value of high-dimensional image-based feature learning in this field [17]. However, most image-based deep learning systems require large annotated image datasets, lesion localization or segmentation procedures, and rigorous validation before routine clinical translation [16,17].

In addition to mammography-based artificial intelligence studies, contrast-enhanced imaging has increasingly been investigated for improving the characterization of suspicious microcalcifications. A recent study proposed a Malignant Calcification Score based on contrast-enhanced mammography descriptors and reported that enhancement-related parameters, including percentage signal difference, modified time–intensity curve, enhancement distribution, and internal enhancement pattern, were independent predictors of malignancy [18]. This score demonstrated higher diagnostic performance than conventional BI-RADS assessments based on mammography and MRI, suggesting that functional enhancement information may provide complementary diagnostic value beyond morphology alone [18]. Recent segmentation- and detection-oriented studies have also demonstrated the potential of automated microcalcification localization, including foundation model-based segmentation, real-time edge-compatible detection algorithms, and deep learning-enabled spectral mammography approaches [19,20,21]. Advanced attention-based deep learning architectures have further shown that wavelet decomposition, feature fusion, and attention mechanisms may improve the detection and classification of subtle mammographic abnormalities [22,23]. Nevertheless, direct image-based deep learning, radiomics, and segmentation workflows remain highly dependent on image quality, annotation consistency, scanner-related variability, and sufficiently large training cohorts. Therefore, in limited retrospective datasets, structured radiological descriptors derived from routine mammography and DCE-MRI interpretation may offer a practical and interpretable alternative for preliminary machine learning-based malignancy prediction.

AI-based radiomic studies have been widely applied in medical practice. Recently developed AI-based computer-aided diagnosis (AI-CAD) software has demonstrated higher diagnostic performance than radiologists in detecting breast cancer by predicting the likelihood of malignancy in breast lesions [24]. In a study on breast microcalcifications, AI-CAD and radiologists demonstrated similar diagnostic performance in characterizing suspicious microcalcifications on mammography [25]. Recent advances in artificial intelligence have further strengthened the role of data-driven approaches in breast imaging. Blahová et al. provided a comprehensive review of neural network-based mammography analysis, emphasizing that data augmentation techniques significantly improve model generalization and diagnostic accuracy in breast cancer detection tasks [26]. Their findings highlight the importance of robust preprocessing strategies in enhancing the reliability of AI-based systems, particularly in limited and imbalanced medical datasets. In addition, Nowakowska et al. proposed an explainable precision medicine framework combining radiomics and deep learning for the classification of contrast agent uptake patterns in breast MRI [27]. Their study demonstrated that integrating handcrafted radiomic features with deep learning representations not only improves classification performance but also enhances model interpretability, which is critical for clinical adoption. These findings further support the growing importance of multimodal and explainable AI approaches in improving diagnostic decision-making in breast imaging. Beyond direct microcalcification analysis, recent studies have also demonstrated the broader applicability of artificial intelligence and machine learning in breast-related imaging workflows. Ünal et al. developed a machine learning-based patient-specific prediction model for estimating breast radiation absorbed dose during chest CT examinations using structured demographic and anatomical variables, supporting the feasibility of tabular clinical-imaging data for individualized radiological prediction [28]. In another recent study, Ünal et al. proposed a YOLO-v11-based deep learning model for ultrasound-based axillary lymph node localization and benign–malignant classification in breast cancer, demonstrating the potential of AI-supported decision systems in breast imaging. These studies further support the growing role of machine learning and deep learning methods in radiological decision support, while also emphasizing the need to match the modelling strategy to the available data type, whether structured descriptors or direct image-based inputs [29].

Radiomics has recently emerged as an important field in medical imaging by enabling the extraction of quantitative features from radiological images, including intensity, shape, texture, and higher-order imaging biomarkers. In breast imaging, radiomics and machine learning approaches have been increasingly used to characterize lesion heterogeneity, improve malignancy prediction, and support precision imaging workflows. Multimodal radiomics, which combines information from different imaging modalities such as mammography, contrast-enhanced mammography, and MRI, may provide complementary diagnostic information by capturing both morphological and functional tissue characteristics. However, radiomics-based workflows generally require reliable lesion segmentation, standardized imaging protocols, reproducible feature extraction, and sufficiently large datasets for model development and validation. These requirements may limit their immediate applicability in small retrospective cohorts. Therefore, structured radiological descriptors routinely obtained during clinical interpretation may represent a practical and interpretable alternative for preliminary machine learning-based risk stratification, particularly when direct image-based radiomic feature extraction is not feasible.

A combined model using mammography and DCE-MRI for predicting the malignancy of breast calcifications has rarely been reported [30]. Although several studies have investigated artificial intelligence, radiomics, and deep learning approaches for breast lesion characterization, studies specifically focusing on BI-RADS 4–5 microcalcifications using combined mammographic descriptors and DCE-MRI enhancement information remain limited. In this context, the present study contributes preliminary evidence regarding the potential value of integrating routinely available mammographic and MRI-derived descriptors for malignancy prediction in this challenging subgroup.

Although deep learning and radiomics-based approaches have shown promising results in breast imaging, their implementation often requires large annotated image datasets, segmentation procedures, extensive computational resources, and external validation. In contrast, structured radiological descriptors routinely reported in clinical practice may provide a practical and interpretable basis for preliminary decision-support models, particularly in limited-data settings. Therefore, the present study aimed to develop and internally evaluate a structured feature-based machine learning framework for distinguishing benign and malignant BI-RADS 4–5 mammographic microcalcifications by combining patient age, mammographic morphology, calcification size, distribution pattern, and DCE-MRI contrast enhancement status. Rather than performing direct image-based radiomics or deep neural network analysis, this study focused on the diagnostic value of clinically available radiological descriptors. The proposed framework is intended as an exploratory decision-support approach, and further validation using larger multicenter cohorts and quantitative imaging biomarkers is required before clinical application.

## 2. Materials and Methods

In this study, a retrospective and data-driven methodological framework was established to investigate the diagnostic value of mammographic microcalcification features and dynamic contrast-enhanced magnetic resonance imaging (DCE-MRI) findings in the differentiation of benign and malignant BI-RADS 4 and BI-RADS 5 lesions. Clinical, radiological, and histopathological data obtained from eligible patients were systematically collected and analyzed. The study design comprised patient selection according to predefined inclusion and exclusion criteria, radiological evaluation of mammographic morphology and calcification distribution patterns, assessment of MRI contrast enhancement characteristics, dataset construction, and machine learning-based classification. To ensure methodological consistency, all radiological variables were categorized according to standardized diagnostic criteria, and the final pathological diagnosis was used as the reference standard. Subsequently, multiple machine learning algorithms were trained and optimized using cross-validation-based hyperparameter tuning in order to determine the most accurate predictive model for malignancy classification.

This study was designed as a structured clinical-radiological feature classification study rather than a direct image-based radiomics or deep learning study. No convolutional neural network architecture, automated lesion segmentation method, or quantitative radiomic feature extraction pipeline was implemented. Instead, the predictive models were trained using predefined clinical and radiological descriptors obtained from mammographic and DCE-MRI evaluations. This design was selected because the available cohort was limited in size and because the primary aim was to assess whether routinely available radiological descriptors could provide preliminary malignancy prediction support in BI-RADS 4–5 microcalcifications. Therefore, the proposed framework should be interpreted as an exploratory machine learning model based on structured radiological descriptors, not as an end-to-end image analysis system.

### 2.1. Ethical Statements

This study was conducted in accordance with the Helsinki Declaration, as updated in 2013. This retrospective study was approved by the Health Research Ethics Committee of İzmir Katip Çelebi University (0507, on 11 September 2025), and the requirement for individual informed consent was waived for the retrospective analysis.

### 2.2. Patient Population

Between March 2023 and October 2025, a comprehensive retrospective review was conducted in the clinical, pathological, and radiological databases of patients with suspicious microcalcifications detected on mammograms at the İzmir Atatürk Training and Research Hospital, Katip Çelebi University.

The inclusion criteria for the study were as follows:

- Patients with suspicious microcalcifications on mammography;
- Patients whose microcalcifications had been histopathologically evaluated;
- Patients who underwent preoperative bilateral breast MRI.

The exclusion criteria were as follows:

- Patients with a history of breast surgery or chemotherapy prior to the MRI examination;
- Patients with incomplete or low-quality MRI images;
- Patients for whom histopathological results were unavailable;
- Patients with low-quality or incomplete mammography images.

The clinicopathological data were obtained directly from the medical record system and pathology reports. Based on the pathological findings of the biopsy specimen, the final histopathological diagnosis was determined as benign or malignant. The patients’ ages were recorded from the database.

During the study period, 80 patients with suspicious mammographic microcalcifications were initially reviewed. After applying the exclusion criteria, 13 patients were excluded because of incomplete or low-quality MRI images, 5 because of unavailable histopathological results, 6 because of low-quality or incomplete mammographic images, and 3 because of a history of breast surgery or chemotherapy before MRI examination. Finally, 53 biopsy-confirmed cases were included in the analysis.

### 2.3. Image Analysis

Mammographic images were acquired using a full-field digital mammography system (Giotto; IMS, Bologna, Italy) in the CC (craniocaudal) and MLO (mediolateral oblique) positions. A breast radiologist with 6 years of experience in breast imaging analysed the characteristics of the suspicious microcalcification without knowing the pathological results. The radiologist classified the size of the suspicious microcalcification into three categories: <10 mm, 10–20 mm, and >20 mm. Based on the fifth edition of BI-RADS, updated in 2013, the radiologist determined its morphology (coarse, amorphous, pleomorphic, fine linear) and distribution (diffuse, regional, segmental, linear, clustered).

All examinations were performed on a Siemens MAGNETOM Lumina (Erlangen, Germany) system with a 3.0 T magnetic field strength, using an 18-channel bilateral breast coil. The dynamic contrast-enhanced examination was conducted using the standard 3D T1 fat-suppressed VIBE/Dixon-VIBE protocol: 1 pre-contrast + at least 5 post-contrast phases were planned, with a targeted temporal resolution of 60–90 s per phase (TR/TE 4–5/1.3–2.0 ms, flip angle 9–12°, isotropic 0.8–1.2 mm). Gadolinium chelate was administered intravenously at a dose of 0.1 mmol/kg with an injection rate of 1.5–2.0 mL/s and washed out with 20 mL of saline; the first post-contrast phase was initiated approximately 15–20 s after the bolus. Pixel-based subtraction from pre-contrast volumes to post-contrast volumes was applied in all post-processing steps; MPR (axial/sagittal/coronal) and MIP reconstructions were generated.

The breast MRI image analyses were performed by a radiologist with over six years of experience in breast MRI. Areas showing calcifications were interpreted as either “with contrast” or “without contrast”. All mammographic and MRI-based descriptors used in the predictive models were derived from radiological interpretation rather than automated image segmentation or direct image-based feature extraction. Mammographic morphology, calcification size, and distribution pattern were evaluated according to BI-RADS-based criteria. DCE-MRI enhancement was recorded as a binary structured variable indicating the presence or absence of visually detectable contrast enhancement corresponding to the mammographic calcification area. Kinetic enhancement curves, quantitative perfusion parameters, detailed MRI BI-RADS descriptors, and time–intensity curve characteristics were not incorporated into the models because these data were not consistently available in the retrospective dataset. Therefore, the DCE-MRI variable should be interpreted as a simplified clinical descriptor rather than a comprehensive quantitative DCE-MRI biomarker. All imaging evaluations were performed by a single breast radiologist; therefore, interobserver agreement analysis could not be performed. This issue is acknowledged as an important limitation of the present study.

**Figure 1:**
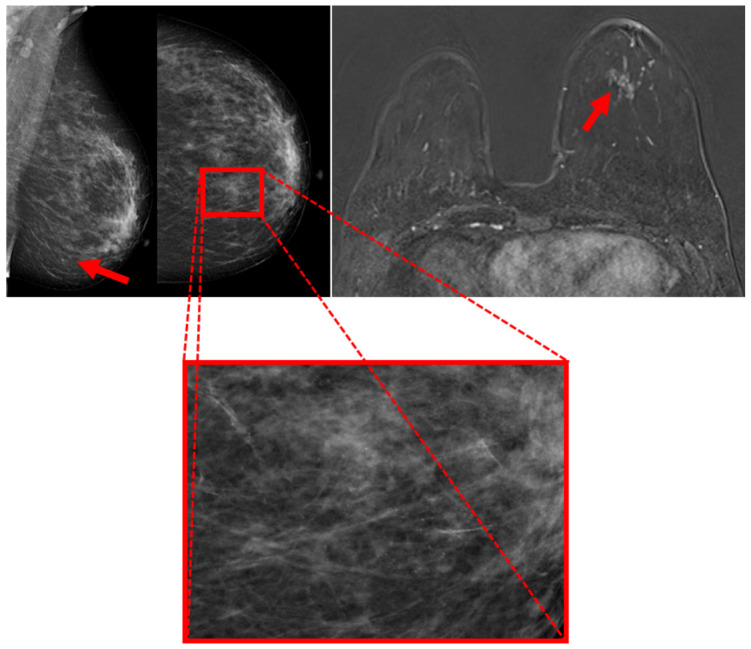
MLO mammogram, CC mammogram, and DCE-MRI images. Red arrows indicate the suspicious microcalcification area on mammographic images, and the red square indicates the corresponding contrast-enhancing area on DCE-MRI. MLO: mediolateral oblique; CC: craniocaudal; DCE-MRI: dynamic contrast-enhanced magnetic resonance imaging.

A pleomorphic, fine, linear, branching calcification area showing a regional distribution pattern in the lower-middle quadrant of the left breast mammogram demonstrates contrast uptake on DCE-MRI. The pathological result for the described calcification area was diagnosed as DCIS. Images from the left MLO mammogram, left CC mammogram, and DCE-MRI are shown in Figure 1.

### 2.4. Dataset Description and Distribution Analysis

The dataset used in this study includes the mammographic morphological characteristics and contrast enhancement patterns on DCE-MRI of suspicious microcalcifications with a known pathological diagnosis detected on mammography.

The main aim of this dataset is to analyse the correlation between imaging results and histopathological biopsy results to design predictive models using machine learning algorithms that can identify benign and malignant lesions of the breast. This dataset has 53 biopsy samples from patients and six variables.

**Figure 2:**
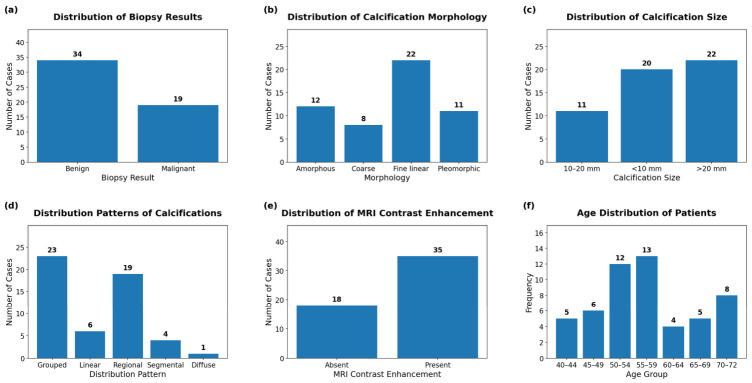
Distribution of dataset variables: (**a**) biopsy outcome, (**b**) calcification morphology, (**c**) calcification size, (**d**) calcification distribution pattern, (**e**) DCE-MRI contrast enhancement status, and (**f**) patient age.

The biopsy result variable is the target variable of the dataset and refers to the histopathological results obtained after conducting biopsy procedures. The biopsy results were represented using a binary classification variable where benign lesions were coded as 0 and malignant lesions were coded as 1. Of the 53 cases in the dataset, 34 cases (64.15%) are represented by benign lesions, while 19 cases (35.85%) are represented by malignant lesions. The distribution of the biopsy results is shown in Figure 2a. The dataset also contains information on the morphology of microcalcifications, which refers to the morphological appearance of microcalcifications detected in mammographic images. Morphological features are among the most significant indicators used in breast cancer diagnosis. In the dataset, morphological features were represented using four categories. Coarse calcifications were represented by 1, amorphous calcifications by 2, pleomorphic calcifications by 3, and fine linear calcifications by 4. The distribution of the morphological features in the dataset is shown in Figure 2b. Another critical attribute that has been incorporated into the dataset is the size of calcification, which represents the approximate diameter of the observed microcalcifications in mammography images. To ensure uniformity in the dataset and make the modelling process easier, the size of microcalcifications was defined as an attribute with three discrete values depending on the diameter of the lesion. Microcalcifications measuring less than 10 mm were assigned the value 1, calcifications measuring between 10 mm and 20 mm were assigned the value 2, and calcifications measuring more than 20 mm were assigned the value 3. The histogram representing the distribution of the size of calcification in the dataset is shown in Figure 2c. Another attribute that has been used in the dataset is the distribution pattern of calcifications. The distribution patterns are commonly employed in breast imaging for evaluating the suspiciousness of calcifications. In the current dataset, distribution patterns were assigned numerical values to denote five distinct diagnostic groups. Diffuse distribution was assigned the value 1, regional distribution was assigned the value 2, segmental distribution was assigned the value 3, linear distribution was assigned the value 4, and grouped distribution was assigned the value 5. The frequency distribution of the calcification patterns is shown in Figure 2d. The current dataset also contains a variable representing the MRI contrast enhancement, which denotes the presence of contrast enhancement noticed in magnetic resonance imaging studies. Contrast enhancement is often a sign of high vascular activity in the breast tissue and is commonly found in malignant tumours. In the current dataset, the presence of contrast enhancement was denoted by value 1, and the absence of contrast enhancement was denoted by value 2. The distribution of cases for MRI contrast enhancement is shown in Figure 2e. One of the variables considered in the dataset is Age, which corresponds to the age of the patient at the time of diagnosis and is presented as a continuous numerical variable. In the dataset that was analysed, the ages of the patients range between 40 and 72 years, with an average age of about 54.36 years. Age is one of the most important demographic variables in breast cancer studies because the chances of malignancy are known to increase with increasing age. The distribution of the ages of the patients in the dataset is shown in Figure 2f, which presents the frequency distribution of the age variable.

Generally, the dataset combines demographic variables with characteristics of radiological images that are commonly used in the diagnosis of breast cancer. The variables offer a complete description of clinical signs used in the assessment of breast lesions and can be used to create machine learning models for predicting biopsy results. The distribution of all variables is shown collectively in Figure 2, which shows the graphical representation of biopsy outcomes (a), calcification morphology (b), calcification size (c), calcification distribution patterns (d), MRI contrast enhancement (e), and patient age (f).

### 2.5. Feature Encoding and Preprocessing

Before model training, the dataset was reviewed for incomplete or unavailable values. Cases with missing histopathological outcomes, insufficient mammographic image quality, or incomplete MRI data had already been excluded according to the predefined exclusion criteria. Patient age was retained as a continuous numerical variable. Calcification morphology, calcification size, distribution pattern, and DCE-MRI enhancement status were included as structured categorical variables based on radiological assessment.

Nominal categorical variables were encoded using integer-based category labels before model development. Calcification morphology, calcification size, distribution pattern, and DCE-MRI enhancement status were represented by predefined numerical codes. No one-hot encoding was applied in the present analysis. Because morphology and distribution categories do not necessarily represent a true biological order, the use of integer-based coding was considered during interpretation and is acknowledged as a methodological limitation, particularly for distance-based and linear models.

To minimize the risk of data leakage during hyperparameter tuning, preprocessing steps were implemented within the cross-validation workflow. Scaling was applied only where required by the algorithm, particularly for Logistic Regression, Support Vector Machine, and K-Nearest Neighbors. Encoding and scaling procedures were fitted on the training folds and then applied to the corresponding validation fold within each cross-validation iteration. No preprocessing step was fitted on the full dataset before cross-validation.

### 2.6. Machine Learning Models Hyperparameter Optimization

The hyperparameter optimization process used for the machine learning algorithms in this study is shown in Table 1. For each of the classification algorithms, a set of hyperparameters was searched using GridSearchCV with cross-validation. The table shows the hyperparameters searched and the best hyperparameters found based on the best F1-score obtained during the validation of the models. The machine learning algorithms used in this study include Logistic Regression, Support Vector Machine with RBF kernel, K-Nearest Neighbours, Decision Tree, Random Forest, Extra Trees, Gradient Boosting, AdaBoost, and CatBoost. The results obtained show that each of the models has a distinct set of hyperparameters that need to be used for optimal performance. For example, the Support Vector Machine classifier performed best with a high regularization parameter (C = 100) and a small gamma value, which is indicative of a complex nonlinear decision boundary. Additionally, ensemble-based models such as Random Forest and Gradient Boosting classifiers performed best with a set of hyperparameters that included the use of multiple estimators and tree depth control. The hyperparameter grids were selected to cover commonly used parameter ranges for each conventional machine learning algorithm while avoiding excessively large search spaces that could further increase overfitting risk in this small dataset.

To ensure the reliability and generalization ability of the machine learning models, a five-fold cross-validation (CV = 5) approach was adopted during the model training and hyperparameter tuning phases. Cross-validation is a popular resampling method in machine learning that enables the effective use of the available dataset for both training and validation tasks, especially when dealing with a small dataset.

In the current study, the dataset was split into five roughly equal portions (folds). In each repetition of the cross-validation procedure, four of the folds were used for training the model, while the other fold was used for validation. This procedure was repeated five times, such that each of the portions was used exactly once as the validation set. The performance of the final model during hyperparameter tuning was calculated by taking the average of the validation results across all five folds. There are several key benefits of using five-fold cross-validation in predictive modelling research. First, it prevents overfitting by testing the model on several data splits instead of using a train and test split. Second, it is a more stable and unbiased measure of model performance by reducing the variance caused by randomly splitting the data. Finally, cross-validation is best used when working with small datasets, like the mammography biopsy dataset in this study, which only has 53 observations. The cross-validation procedure enables the models to discover more robust patterns in the data while still having a fair evaluation process.

Five-fold cross-validation was used to make efficient use of the limited dataset and to obtain an internal estimate of model performance during hyperparameter tuning. In each iteration, the training and validation folds were separated within the cross-validation procedure, and model performance was averaged across folds. However, cross-validation does not fully eliminate the risk of overfitting, particularly in small datasets and when multiple algorithms and hyperparameter combinations are evaluated. Therefore, the reported performance metrics should be interpreted as internally validated exploratory results rather than definitive evidence of clinical generalizability. External validation in larger independent cohorts is required to confirm the robustness, reproducibility, and clinical utility of the proposed models.

To quantify the uncertainty associated with the limited test-set size, approximate 95% confidence intervals were calculated for the main performance metrics using the observed test-set confusion matrix counts. Wilson score intervals were used for accuracy, precision, and recall. The uncertainty of the F1-score was estimated using bootstrap resampling of the observed confusion-matrix cell counts with 1000 iterations. ROC-AUC confidence intervals were approximated using the number of benign and malignant cases in the test set. These confidence intervals were used to support a cautious interpretation of model performance and should be interpreted in light of the small test-set size.

## 3. Results

This section will discuss experimental results obtained using machine learning models developed to classify the pathology results of suspicious microcalcifications detected on mammograms.

After the hyperparameter optimization process was completed, as described in the previous section, the models were tested using a variety of metrics to evaluate the classification ability of the models. The metrics used to evaluate the models include accuracy, precision, recall, F1-score, ROC-AUC, and log loss. The experimental analysis focuses on comparing the performance of different machine learning algorithms on a dataset of suspicious microcalcifications in mammography.

The research uses a set of machine learning classifiers, such as Logistic Regression, Support Vector Machine (with RBF kernel), K-Nearest Neighbours, Decision Tree, Random Forest, Extra Trees, Gradient Boosting, AdaBoost, and CatBoost. The aim is to find out which of these algorithms produces the most accurate results in terms of distinguishing benign from malignant lesions of the breast. The results of these algorithms on the suspicious microcalcifications on the mammography dataset are presented in Table 2.

The confidence intervals were wide for several metrics, reflecting the limited number of test cases. Therefore, the point estimates reported in Table 2 and the confidence intervals reported in Table 3 should be interpreted cautiously as internal exploratory performance results rather than stable estimates of external model performance.

The performance metrics show that Logistic Regression achieved the highest point estimates among the evaluated models, with an accuracy of 0.909, an F1-score of 0.889, and a ROC-AUC of 1.000 in the internal evaluation. However, because the dataset included only 53 cases and no independent external validation cohort was available, the ROC-AUC value of 1.000 should be interpreted cautiously. This result may reflect the separability of the current dataset, but it may also be influenced by sampling variability and model instability associated with small sample sizes. Therefore, the apparent superiority of Logistic Regression should be regarded as preliminary until confirmed by larger validation cohorts and appropriate statistical comparison tests. AdaBoost also showed favorable sensitivity-oriented performance, achieving an accuracy of 0.818, an F1-score of 0.800, and a recall of 1.000 in the internal evaluation, suggesting that it correctly identified all malignant cases in the evaluated split. Nevertheless, this finding should also be interpreted with caution because of the limited number of malignant cases. The Support Vector Machine with radial basis function kernel showed a balanced performance profile, with an accuracy of 0.818, precision of 0.750, recall of 0.750, F1-score of 0.750, and ROC-AUC of 0.857. In contrast, K-Nearest Neighbors, Decision Tree, Random Forest, Gradient Boosting, and CatBoost demonstrated moderate internal performance, while Extra Trees produced the lowest F1-score among the evaluated models. Overall, these findings suggest that simpler linear or sensitivity-oriented ensemble models may perform favorably in this structured feature-based dataset; however, the differences between classifiers should be interpreted descriptively rather than as statistically confirmed superiority. Because the dataset showed a moderate class imbalance, with 34 benign and 19 malignant cases, precision and F1-score should be interpreted cautiously. In a small test set, even one or two false-positive or false-negative classifications can substantially affect these metrics. Therefore, the reported precision and F1-score values should be regarded as descriptive internal performance estimates rather than stable indicators of external model performance.

**Figure 3:**
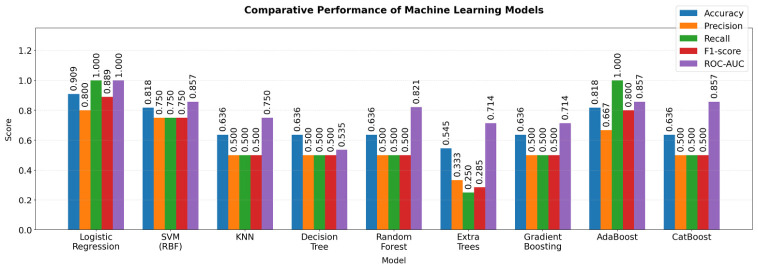
Comparative internal performance metrics of the evaluated machine learning models for benign–malignant classification of BI-RADS 4–5 microcalcifications.

Figure 3 illustrates the relative performance of the machine learning models tested in this study. The performance of the models is measured using a variety of metrics in classification tasks, such as accuracy, precision, recall, F1-score, and ROC-AUC. These metrics help in determining the capacity of the models to classify the biopsy outcomes as either benign or malignant.

Although Figure 3 visually summarizes the comparative performance of the evaluated classifiers, the differences between models should be interpreted descriptively rather than as statistically confirmed superiority. Because no formal statistical comparison test was performed and the number of test cases was limited, the graphical comparison primarily provides an exploratory overview of internal model behavior.

**Figure 4:**
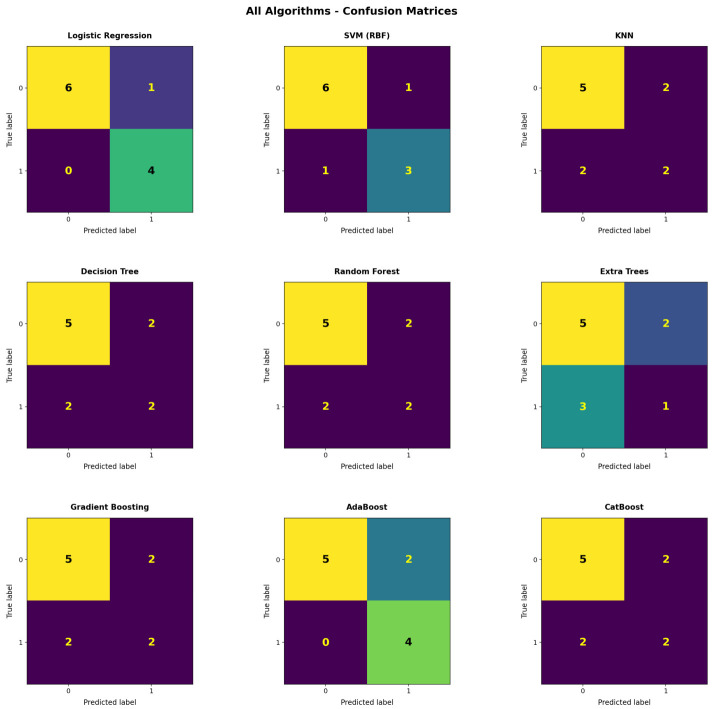
Confusion matrices of machine learning models evaluated for the pathological classification of suspicious microcalcifications detected on mammograms. Class 0 represents benign lesions, and class 1 represents malignant lesions. In the color scale, darker blue/purple tones indicate lower cell counts, green tones indicate intermediate cell counts, and brighter yellow tones indicate higher cell counts.

Figure 4 shows the confusion matrices of the machine learning algorithms used in this study to classify the pathology results of suspicious microcalcifications detected on mammograms. The confusion matrix is a table used to describe the performance of a classification algorithm; it shows the number of true and false positives and negatives. In this study, class 0 is used to represent benign lesions, while class 1 is used to represent malignant lesions. The confusion matrix shows the number of true positives (TP), true negatives (TN), false positives (FP), and false negatives (FN).

From a clinical perspective, false-negative classifications are particularly important because they may delay the diagnosis of malignant lesions. In contrast, false-positive classifications may increase unnecessary biopsy recommendations and patient anxiety. Therefore, the interpretation of confusion matrices should consider not only overall accuracy but also the balance between sensitivity and specificity. In the present exploratory analysis, Logistic Regression and AdaBoost showed favorable sensitivity-oriented behavior; however, the small number of malignant cases limits the reliability of these observations.

The confusion matrix of the Logistic Regression model shows that it has the best classification results among all the tested models. The model was able to correctly classify six benign samples and four malignant samples, while only one benign sample was misclassified as malignant, and no malignant samples were misclassified as benign. The outcome of the study shows that the Logistic Regression model has a high degree of discrimination and can effectively reduce the occurrence of clinically significant false negatives. The Support Vector Machine (RBF) model also has a high degree of classification accuracy. The confusion matrix shows that the model was able to correctly classify six samples as benign and three as malignant, with errors in the classification of one benign and one malignant sample. Although its performance is slightly less than that of the Logistic Regression model, the SVM model still has a good trade-off between sensitivity and specificity. The AdaBoost model proves to be highly accurate in the clinical diagnostic scenario. The confusion matrix shows perfect identification of malignant instances (four true positives) without any false negatives. The outcome shows that there are two benign samples that were wrongly classified as malignant, indicating a slightly higher false positive rate. In the context of clinical decision support systems, the bias may be acceptable if the aim is to obtain a correct classification of malignant specimens rather than to optimize the reduction in false positives in a particular clinical setting. The performance of other algorithms, namely, K-Nearest Neighbours, Decision Tree, Random Forest, Gradient Boosting, and CatBoost, is of moderate accuracy in classification. Although these algorithms are capable of correctly classifying certain samples as benign or malignant, they show a relatively higher tendency to make mistakes compared with the best-performing algorithms, indicating a higher tendency towards both false positives and false negatives and thus influencing their accuracy compared with the AdaBoost algorithm. Of all the algorithms considered, the Extra Trees classifier had the lowest accuracy in classification. The confusion matrix indicates that there are several malignant samples that are incorrectly classified as benign, indicating the presence of false negatives, which may pose a potential risk in the clinical setting due to the possibility of the presence of malignancies going undetected. The confusion matrices are important in understanding the ability of each algorithm to correctly distinguish between benign and malignant lesions. The experimental outcome suggests that the highest accuracy in malignant classification is obtained by the Logistic Regression and AdaBoost algorithms.

## 4. Discussion

In this study, we internally evaluated several conventional machine learning models for predicting the pathological outcomes of BI-RADS 4–5 suspicious mammographic microcalcifications using structured clinical and radiological descriptors. The evaluated variables included patient age, mammographic morphology, calcification size, distribution pattern, and DCE-MRI contrast enhancement status. Among the tested models, Logistic Regression achieved the highest internal point estimates, with an accuracy of 0.909, an F1-score of 0.889, and a ROC-AUC of 1.000. However, this result should be interpreted cautiously because the dataset was small, single-centered, and lacked an independent external validation cohort. Although the findings suggest that routinely available radiological descriptors may contain useful information for malignancy risk stratification, the reported performance should be regarded as preliminary and exploratory rather than definitive evidence of clinical generalizability. Approximately 80–90% of DCIS cases are associated with microcalcifications, and in 50% of breast cancers, microcalcifications are the only finding detectable on mammography [6,30]. Furthermore, the majority of microcalcifications are found to be benign following diagnostic interventional procedures [6]. The radiologist’s experience is crucial in the detection, accurate interpretation, and determination of the appropriate follow-up protocol for microcalcifications [31]. The mammographic features described in the radiologist’s report guide the clinician following the detection of microcalcifications. Numerous studies have demonstrated that MRI can serve as a guiding tool in the management of microcalcifications detected on mammography [32]. Nevertheless, microcalcifications present diagnostic challenges for both radiologists and clinicians, and their diagnosis typically relies on interventional procedures.

Recently, various computer-aided diagnosis studies have been developed using mammography images for the assessment of microcalcifications. Cai H et al. developed a CNN model for the detection, analysis, and classification of microcalcifications in mammography images. By utilizing filtered deep features fully leveraged by the CNN architecture, a classification accuracy of 89.32% and a sensitivity of 86.89% were achieved [33]. In their study focusing exclusively on BI-RADS 4 microcalcifications, Stelzer et al. obtained an AUC-ROC value of 0.82–0.83 [34]. Marathe et al. analysed amorphous calcifications. By extracting radiomic features from foreground and background masks and global features from extended foreground masks, they achieved an AUC-ROC of 0.73, a sensitivity of 1.0, and a specificity of 0.35 [35]. In a study conducted by Liu et al., the researchers developed a combined deep learning model incorporating mammographic and clinical variables for BI-RADS 4 microcalcifications reviewed by radiologists. When comparing the performance of the combined model with that of breast radiologists in predicting the malignancy of breast microcalcifications, the combined model was shown to have diagnostic capabilities nearly equivalent to those of a senior radiologist [7]. Lei C et al. examined only BI-RADS category 4 microcalcifications on mammograms in a radiomics-based study; in the validation cohort, an AUC of 0.80, a PPV of 73.53, and an NPV of 84.21 were obtained. It was reported that the developed radiomics model performed better than the radiologists’ experience-based prediction model [36]. Studies using combined models for breast microcalcifications are limited in the literature. A combined model developed by Zhao et al. using mammography and MRI examinations of solid breast masses demonstrated higher accuracy compared with radiomic analyses of mammography or MRI images alone [37]. In a radiomic model evaluated by Chen et al. using mammography and MRI examinations that assessed only microcalcifications, the performance of the combined model was shown to be superior to that of mammography and MRI alone. However, this study evaluated BIRADS 3–5 microcalcifications. In our study, we evaluated BIRADS 4–5 microcalcifications, which share similar morphological features and represent the most challenging patient population in daily clinical practice [30].

Unlike direct image-based radiomics or deep learning models, the present framework was based on structured descriptors that are routinely available from radiological interpretation. This design may provide a practical and interpretable approach in limited-data settings, particularly when annotated image datasets or segmentation-based radiomic pipelines are not available. Nevertheless, the model should not be interpreted as a replacement for radiologist assessment or as a clinically deployable decision tool at this stage. Rather, it may be considered a preliminary decision-support framework that could complement radiological interpretation after further validation. Larger multicenter studies, external validation cohorts, radiologist performance comparisons, calibration analyses, and decision curve analyses are required before any conclusions can be drawn regarding its ability to reduce unnecessary biopsies or surgical procedures in clinical practice.

This study has several important limitations. First, the dataset was obtained from a single center and included only 53 cases, including 19 malignant lesions. This limited sample size increases the risk of overfitting and restricts the statistical power and generalizability of the findings. Second, only internal five-fold cross-validation was performed, and no independent external validation cohort was available. Therefore, the reported performance values, particularly the ROC-AUC of 1.000 for Logistic Regression, should be interpreted cautiously. Third, the study did not perform direct image-based radiomics, automated microcalcification segmentation, or deep learning analysis. Instead, the models were trained using structured radiological descriptors manually assessed by a single radiologist. Fourth, interobserver variability could not be evaluated because the imaging features were not independently reviewed by multiple radiologists. Fifth, DCE-MRI information was simplified into a binary contrast-enhancement variable, and kinetic enhancement curves, quantitative perfusion parameters, detailed MRI BI-RADS descriptors, and time–intensity curve characteristics were not included. Sixth, although several machine learning algorithms were compared, formal statistical comparison tests, calibration analysis, decision curve analysis, and radiologist-only performance comparison were not performed. Finally, scanner variability, selection bias, and the retrospective design may have influenced the results. These limitations indicate that the proposed framework should be regarded as an exploratory model requiring further validation rather than a ready-to-use clinical decision tool.

## 5. Conclusions

This study developed and internally evaluated a structured feature-based machine learning framework for differentiating benign and malignant BI-RADS 4–5 mammographic microcalcifications using patient age, mammographic descriptors, and DCE-MRI contrast enhancement status. Among the evaluated conventional machine learning algorithms, Logistic Regression achieved the highest internal performance, with an accuracy of 0.909, an F1-score of 0.889, and a ROC-AUC of 1.000. Nevertheless, these findings should be interpreted with caution because of the limited sample size, single-center retrospective design, and absence of external validation. The results suggest that routinely available radiological descriptors may have potential value for preliminary malignancy risk stratification in suspicious microcalcifications. However, larger multicenter studies incorporating external validation, radiologist performance comparison, calibration analysis, decision curve analysis, and quantitative radiomic or deep learning-derived imaging biomarkers are needed before clinical implementation. In this context, the proposed framework should be considered an exploratory decision-support approach rather than a clinically deployable model.

## Figures and Tables

**Table 1:** Hyperparameter search space and optimal model parameters. SVM (RBF): support vector machine with radial basis function kernel; KNN: K-nearest neighbors.

Model	Param Grid	Best Params
**Logistic Regression**	{“model__C”: [0.01, 0.1, 1, 10, 100], “model__solver”: [“liblinear”, “lbfgs”]}	{“model__C”: 0.01, “model__solver”: “liblinear”}
**SVM (RBF)**	{“model__C”: [0.1, 1, 10, 100], “model__gamma”: [“scale”, 0.1, 0.01, 0.001], “model__kernel”: [“rbf”]}	{“model__C”: 100, “model__gamma”: 0.01, “model__kernel”: “rbf”}
**KNN**	{“model__n_neighbors”: [3, 5, 7, 9], “model__weights”: [“uniform”, “distance”], “model__metric”: [“minkowski”, “euclidean”, “manhattan”]}	{“model__metric”: “minkowski”, “model__n_neighbors”: 9, “model__weights”: “distance”}
**Decision Tree**	{“model__max_depth”: [null, 3, 5, 7, 10], “model__min_samples_split”: [2, 4, 6, 8], “model__min_samples_leaf”: [1, 2, 3, 4], “model__criterion”: [“gini”, “entropy”]}	{“model__criterion”: “entropy”, “model__max_depth”: 5, “model__min_samples_leaf”: 1, “model__min_samples_split”: 2}
**Random Forest**	{“model__n_estimators”: [100, 200, 300], “model__max_depth”: [null, 3, 5, 7, 10], “model__min_samples_split”: [2, 4, 6], “model__min_samples_leaf”: [1, 2, 3]}	{“model__max_depth”: 3, “model__min_samples_leaf”: 1, “model__min_samples_split”: 2, “model__n_estimators”: 200}
**Extra Trees**	{“model__n_estimators”: [100, 200, 300], “model__max_depth”: [null, 3, 5, 7, 10], “model__min_samples_split”: [2, 4, 6], “model__min_samples_leaf”: [1, 2, 3]}	{“model__max_depth”: null, “model__min_samples_leaf”: 1, “model__min_samples_split”: 2, “model__n_estimators”: 100}
**Gradient Boosting**	{“model__n_estimators”: [50, 100, 150, 200], “model__learning_rate”: [0.01, 0.05, 0.1, 0.2], “model__max_depth”: [2, 3, 4]}	{“model__learning_rate”: 0.01, “model__max_depth”: 3, “model__n_estimators”: 200}
**AdaBoost**	{“model__n_estimators”: [50, 100, 150, 200], “model__learning_rate”: [0.01, 0.05, 0.1, 0.5, 1.0]}	{“model__learning_rate”: 0.05, “model__n_estimators”: 150}
**CatBoost**	{“model__iterations”: [100, 200, 300], “model__depth”: [3, 4, 5, 6], “model__learning_rate”: [0.01, 0.05, 0.1], “model__l2_leaf_reg”: [1, 3, 5, 7]}	{“model__depth”: 4, “model__iterations”: 100, “model__l2_leaf_reg”: 7, “model__learning_rate”: 0.1}

**Table 2:** Comparative performance evaluation of machine learning models for suspicious microcalcifications on mammography classification.

Model	Accuracy	Precision	Recall	F1_Score	ROC_AUC	Log_Loss
**Logistic Regression**	0.909	0.800	1.000	0.889	1.000	0.603
**SVM (RBF)**	0.818	0.750	0.750	0.750	0.857	0.466
**KNN**	0.636	0.500	0.500	0.500	0.750	0.563
**Decision Tree**	0.636	0.500	0.500	0.500	0.535	12.64
**Random Forest**	0.636	0.500	0.500	0.500	0.821	0.411
**Extra Trees**	0.545	0.333	0.250	0.285	0.714	0.793
**Gradient Boosting**	0.636	0.500	0.500	0.500	0.714	0.590
**AdaBoost**	0.818	0.667	1.000	0.800	0.857	0.456
**CatBoost**	0.636	0.500	0.500	0.500	0.857	0.506

**Table 3:** Approximate 95% confidence intervals for the main performance metrics based on the observed test-set results. ROC-AUC: receiver operating characteristic–area under the curve; CI: confidence interval.

Model	Accuracy (95% CI)	Precision (95% CI)	Recall (95% CI)	F1-Score (95% CI)	ROC-AUC (95% CI)
**Logistic Regression**	0.909 (0.623–0.984)	0.800 (0.376–0.964)	1.000 (0.510–1.000)	0.889 (0.500–1.000)	1.000 (1.000–1.000)
**SVM (RBF)**	0.818 (0.523–0.949)	0.750 (0.301–0.954)	0.750 (0.301–0.954)	0.750 (0.000–1.000)	0.857 (0.593–1.000)
**KNN**	0.636 (0.354–0.848)	0.500 (0.150–0.850)	0.500 (0.150–0.850)	0.500 (0.000–0.857)	0.750 (0.422–1.000)
**Decision Tree**	0.636 (0.354–0.848)	0.500 (0.150–0.850)	0.500 (0.150–0.850)	0.500 (0.000–0.857)	0.535 (0.164–0.906)
**Random Forest**	0.636 (0.354–0.848)	0.500 (0.150–0.850)	0.500 (0.150–0.850)	0.500 (0.000–0.857)	0.821 (0.531–1.000)
**Extra Trees**	0.545 (0.280–0.787)	0.333 (0.061–0.792)	0.250 (0.046–0.699)	0.286 (0.000–0.667)	0.714 (0.372–1.000)
**Gradient Boosting**	0.636 (0.354–0.848)	0.500 (0.150–0.850)	0.500 (0.150–0.850)	0.500 (0.000–0.857)	0.714 (0.372–1.000)
**AdaBoost**	0.818 (0.523–0.949)	0.667 (0.300–0.903)	1.000 (0.510–1.000)	0.800 (0.400–1.000)	0.857 (0.593–1.000)
**CatBoost**	0.636 (0.354–0.848)	0.500 (0.150–0.850)	0.500 (0.150–0.850)	0.500 (0.000–0.857)	0.857 (0.593–1.000)

## Data Availability

The data that support the findings of this study are not publicly available due to ethical and privacy restrictions related to patient confidentiality. Anonymized data may be obtained from the corresponding author upon reasonable request.
